# Incidence of Clinically Significant Aniseikonia Following Encircling Scleral Buckle Surgery: An Evaluation of Refractive and Axial Length Changes Requiring Intervention

**DOI:** 10.3390/vision5010007

**Published:** 2021-02-03

**Authors:** Craig Wilde, Mary Awad, Gavin Orr, Dharmalingam Kumudhan, Saker Saker, Anwar Zaman

**Affiliations:** Ophthalmology Department, EENT Centre, Queen’s Medical Centre, B Floor, Nottingham NG7 2UH, UK; maryawad@nhs.net (M.A.); gavin.orr@nuh.nhs.uk (G.O.); sightsaver@gmail.com (D.K.); saker.saker@nuh.nhs.uk (S.S.); anwar.zaman@nuh.nhs.uk (A.Z.)

**Keywords:** aniseikonia, encirclement, scleral buckle, anisometropia, pars plana vitrectomy, retinal detachment, axial length, keratometry

## Abstract

To evaluate the incidence of symptomatic anisometropia and aniseikonia requiring intervention following surgery with combined pars plana vitrectomy (PPV) and broad 276 style encircling scleral buckle (ESB) for the repair of rhegmatogenous retinal detachments (RRD) and to report axial length (AL) and keratometry changes, a retrospective review of consecutive RRD patients treated with combined PPV and ESB between June 2016 until September 2019 was performed. All patients with symptomatic optically induced aniseikonia requiring additional interventions or surgical procedures including clear lens exchanges, secondary intraocular lens implants or contact lenses were documented. Keratometry and AL measurements were recorded for each eye and changes calculated. In total, 100 patients underwent combined PPV, ESB and endotamponade with mean age of 59.47 years (SD 11.49). AL was significantly increased (25.39 mm [SD 1.27] to 26.54 mm [SD 1.16], *p* = 0.0001), with a mean change of 1.15 mm (SD 0.67). Mean corneal astigmatism increased by –0.95 D (SD 0.51) in control eyes preoperatively and –1.33 (SD 0.87) postoperatively (*p* = 0.03). Over half of phakic patients (39/61; 64%) developed a visually significant cataract, subsequently undergoing surgery. Six of 100 patients developed symptomatic anisometropia with aniseikonia postoperatively (6%). Four proceeded with clear lens exchange despite absence of visually significant cataract (4%). Two of these initially trialled contact lenses (2%). One was intolerant, while the other decided to proceed with clear lens exchange for convenience. Only one patient (1%), being pseudophakic in both eyes, had persistent anisometropia/aniseikonia. AL and keratometry changes induced by encirclement with broad solid silicone rubber buckles are acceptable and similar to those reported previously using narrow encircling components, being unlikely to induce troublesome symptomatic anisometropia/aniseikonia. Many patients are phakic and develop visually significant cataracts, allowing correction of changes induced with the aim of visual restoration. A minority require more prolonged methods of visual rehabilitation, such as contact lens wear or clear lens exchanges. Caution and appropriate consent should be made in patients that are pseudophakic in both eyes at presentation.

## 1. Introduction

Scleral buckle (SB) use with or without encirclement, combined with pars plana vitrectomy (PPV) remains controversial. The rationale, benefits, disadvantages and indications are often debated [1,2,3,4,5]. Many studies are non-randomised and retrospective, with inherent selection bias regarding treatment assignment. Studies often fail to record prevalence of early (grade A/B) proliferative vitreoretinopathy (PVR) among different treatment arms, making comparisons difficult [2]. A recent randomised controlled trial (RCT) demonstrated the advantage of additional encirclement in eyes with inferior breaks [6]. An older RCT suggested benefit for anatomical success among pseudophakic eyes with additional SB use during PPV [7]. Surgical techniques chosen for RRD repair remain highly variable, based not only on patient and ocular factors, but experience and preferences of surgeons along with equipment availability.

Although SB popularity appears to have declined, they are still commonly undertaken as reflected in the ‘National Electronic Retinal Detachment Surgery Audit: Feasibility Report’ in the UK, where 507 of 10,054 (5.0%) primary operations included combined PPV and SB [8]. Buckling has several distinctive potential complications in addition to PPV including infection, exposure, extrusion, diplopia and increased incidence of choroidal haemorrhage, among others [9,10,11,12]. One further issue includes postoperative shifts in refraction by globe indentation, generally inducing myopia from elongation of axial length (AL) [13,14,15,16,17]. Corneal curvature can also alter [18,19]. Disparity among published results may reflect different surgical techniques, particularly the degree of encirclement tightening. Some studies include only solid silicone rubber SB, without additional PPV [20], while others report changes with silicone sponges [21]. Some use traditional 1.5 mm or 2 mm narrow encircling bands in addition to localised buckles [20,22], with the position of the encirclement varying from equatorial to more anterior. Some older studies use scleral dissection and other techniques no longer in use [17]. Often methods to tighten encircling bands are vague, being described as enough to induce ‘moderate equatorial indentation,’ which is subjective and dependent on other variables, including the degree of subretinal fluid drainage, which could lead to capricious results [20]. Others report no details regarding methods of scleral encirclement, including the size or degree of shortening [23], making meaningful comparisons difficult. Older publications use A-scan ultrasound to measure AL, which can be less accurate, particularly in macular involving retinal detachments, underestimating preoperative AL. Oftentimes, case series are small, limiting detection of extreme changes. Older publications include large subgroups of aphakic eyes, making results less generalisable to modern populations [24].

It is likely the most widely used buckling technique utilises segmental circumferential solid silicone explants, with or without additional narrow silicone encirclement. To the best of our knowledge, AL and keratometry changes induced by 360-degree encirclement with broad solid silicone explants combined with PPV remains unpublished. This technique is mostly utilised in our department when ESB is combined with PPV.

Final refractive outcome following PPV and ESB is multifactorial, depending on interplay between AL changes, lens status, induced lenticular myopia and need for subsequent cataract surgery. Published literature focuses primarily on changes in biometric measurements. To the best of our knowledge incidence of clinically significant anisometropia necessitating unplanned clinical interventions, including clear lens exchanges or initiation of contact lens wear, has not been documented. With this in mind, we sought to determine the frequency of such outcomes. This will allow appropriate consenting of our future patients, better informing them of risk. This document provides new and novel information from a retinal detachment population that can be used for appropriate preoperative consenting purposes.

## 2. Materials and Methods

This study includes a large retrospective review of all consecutive patients with RRD assessed in our department (Queen’s Medical Centre, Nottingham, UK) who underwent surgery involving combined PPV, ESB and endotamponade between June 2016 and September 2019. All historical ophthalmic operation lists within our hospital were manually searched (by an ophthalmologist—M.A.), on a list-by-list basis, to identify a retrospective cohort of patients who had undergone combined PPV and ESB surgery. To optimise case identification, the initial theatre list search strategy was broader, including all patients who were scheduled for combined PPV and ESB or buckle. This was done in an effort to identify any patients who were erroneously enumerated as a segmental buckle rather than ESB during the theatre listing process. Subsequent operation note review was undertaken to ensure only ESB patients were included within the final cohort. Both macular-on and -off cases were included. Once identified, all available patient records, including written and electronic notes within our hospital, were reviewed (by an ophthalmologist—M.A.) in a longitudinal fashion from presentation until discharge. All clinical entries across each note platform used within our trust, including scanned copies of hand written notes (Digital Health Records) and purely digital entries (Medisoft), were reviewed. Data extraction was simultaneously undertaken using an Excel spreadsheet. Data regarding cataract surgery undertaken elsewhere or information regarding delayed re-presentation within other institutions following initial discharge from our hospital were not analysed, given the retrospective nature of our study and ethical considerations.

Written informed consent for surgery was gained from all participants and study conducted in accordance with the Declarations of Helsinki. Exclusion criteria from analysis of AL measurement or refractive outcome were eyes with failure of anatomical success and those who had previously undergone contralateral PPV and ESB surgery.

All ESB and combined PPV procedures were performed by two vitreoretinal surgery consultants (A.Z. or C.W.) or vitreoretinal surgery trainees under direct supervision using the same surgical technique under general anaesthetic or following peribulbar local anaesthetic block. Initial 360° conjunctival peritomy was performed. Rectus muscles were isolated and slung with 2-O silk. Subsequent 23G PPV using the one step entry site alignment valved cannula with the Stellaris PC System (Bausch and Lomb) or EVA Phaco-Vitrectomy System (DORC) was performed. A non-contact wide-angle viewing system (BIOM; Oculus, Wetzlar, Germany) was used for posterior segment examination. Surgical technique variations, including heavy liquid, extent and use of endolaser or trans-scleral cryotherapy for retinopexy were made according to clinical need and intraoperative findings. Fluid-air exchange was performed with internal drainage of sub-retinal fluid. Encirclement using a 276 style-solid silicone tire (7 mm wide) was undertaken with buckle being placed 360° around the globe under the four rectus muscles. One horizontal 5–0 ethibond mattress placement suture was used within each quadrant, securing the tire in position. Sutures were passed 1 mm behind the posterior margin of the tire, while anterior bites were placed level with the rectus muscle insertions. To achieve the desired indentation, the band was resected at the lateral border of insertion of the superior rectus and superior border of the lateral rectus muscles. Buckle ends were approximated and sutured together using two 5–0 ethibond. Where gas endotamponade was required, an isovolumetric concentration (12%) of perfluoropropane (C_3_ F_8_) was used. When deemed necessary, silicone oil (1000 cs) was used, for indications including proliferative vitreoretinopathy (PVR) or one-eyed patients. Filling was achieved via a three-way tap and oil pump. Internal examination using the BIOM was undertaken, ensuring appropriate indentation was visible and endotamponade fill performed. No additional radial buckles or silicone sponges were placed.

Postoperative review was arranged on day 1, then at week 1, 3 and 7, with further follow up in a longitudinal fashion as indicated with full clinical evaluation. All patients underwent pre- and postoperative best corrected LogMAR visual acuity (BCVA) testing, mydriatic slit lamp examination including fundus review and intraocular pressure (IOP) assessments.

Medical records were reviewed for surgical details, preoperative lens status and patient demographic data. Clinically important postoperative findings were recorded including the development of visually significant cataracts requiring surgery, onset of new refractive errors that caused symptomatic anisometropia and required additional steps to achieve visual rehabilitation prior to discharge including contact lens wear, clear lens exchange in either eye or secondary intraocular lens implant/piggy back lens placement.

Where available pre- and postoperative refractive errors were recorded. When clinically indicated postoperative biometry was performed with the IOLMaster (Carl Zeiss Meditec, Jena, Germany). Axial length (AL) and Keratometry (K) readings were recorded for both eyes. Contralateral eyes were used as a control group for comparison. If AL could not be measured optically with the IOLMaster, because of dense cataracts, then measurements with A-scan ultrasound were taken using the Nidek (Gamagori, Japan). Contralateral eyes that had previously undergone ESB were excluded from analysis.

Notes were reviewed for symptoms suggestive of clinically significant aniseikonia (in the presence of anisometropia), including reports of difficulty reading, especially improved by closing one eye, new onset diplopia (not associated with restriction of ocular motility), reports of new vertigo/dizziness, reported disrupted space perception/loss of stereopsis or intolerance of new spectacles secondary to anisometropia. We highlight that this is a retrospective study of data collected through routine clinical practice. All clinical entries within notes and tests undertaken, such as postoperative biometry or refraction, were felt to be clinically indicated at the time to have been performed and requested by the attending ophthalmologist, who would have been either a vitreoretinal surgery consultant or fellow or a trainee under supervision. Enquiries and documentation of the relevant symptoms and statements above regarding differentiation between optical (secondary to induced anisometropia) or retinal aniseikonia (secondary to a retinal fold or epiretinal membrane {ERM}) was also performed as part of patients’ routine clinical care at the time of the attendance and documented in the notes accordingly.

Available biometrics (AL and K-readings) were compared between operated eyes and contralateral/un-operated eyes. The data were expressed as mean ± standard deviation (SD) for operated and control eyes. Data distribution was assessed for normality using the Shapiro–Wilk and Kolmogorov Smirnov tests. Comparisons were made using paired t-test, with a *p*-value ≤ 0.05 being considered statistically significant. Data analysis was conducted using Statistical Package for the Social Sciences Mac Version 24 (SPSS, Inc., Chicago, IL, USA) software.

## 3. Results

In total 100 patients met inclusion criteria, undergoing combined PPV, ESB and endotamponade. Mean age was 59.47 years (SD 11.49) with a range of 28–84 years (interquartile range 14.5 years). Most eyes (74/100; 74%) presented with macular-off RRD with the majority being phakic at presentation in their operated eye (61/100; 61%). At presentation 2 out of 61 phakic eyes (3.28%) had a pre-existing cataract that was removed during RRD surgery. Postoperatively, a further 39 (64.0%) developed or subsequently re-presented with visually significant cataracts and had surgery. One patient was left aphakic with subsequent secondary sulcus IOL. Four of 61 phakic patients later had a clear lens exchange to overcome induced symptomatic anisometropia (6.6%).

The mean follow-up period after surgery until discharge or last clinic appointment was 469 days (SD 351), with a range from 0–1570 days (interquartile range 486). Follow-up duration varied considerably depending on factors, including whether patients underwent subsequent cataract surgery or developed ocular hypertension/glaucoma that required ongoing treatment. Only two patients from within the surgical cohort were lost to follow up within the first two months following surgery. One further patient preferred ongoing visits privately outside the NHS environment. Otherwise, all other patients attended.

Biometry data and AL measurements were available for 35 eyes. Two of these were excluded from analysis as they had previous contralateral eye encirclements. Mean AL increased from 25.39 mm (SD 1.27; 95% CI 24.94–25.83) preoperatively to 26.54 mm (SD 1.16; 95% CI 26.16–26.91) postoperatively (*p* = 0.0001), as summarised in Table 1. Mean change in AL was 1.15 mm (SD 0.67; 95% CI 0.91–1.39). Of the 34 eyes with available biometry, only 1 (2.9%) demonstrated no change in AL, while the remainder increased.

Mean corneal astigmatism in control eyes was –0.95 D (SD 0.51; 95% CI −0.78 to −1.13) and postoperatively was −1.33 D (SD 0.87; 95% CI −1.04 to −1.61). The difference was statistically significant (*p* = 0.03). Mean change in corneal astigmatism was –0.41 D (SD 0.86; 95% CI −0.11 to −0.71). Keratometry showed value changes in corneal astigmatism ranging from a decrease of 0.59 dioptres (D) to an increase of 3.57 D. Eleven of 34 eyes (32.4%) demonstrated a reduction in corneal astigmatism following encirclement with the remainder showing an increase. Five out of 34 patients had an increase in astigmatism of magnitude 1.00 D or greater (14.7%). No patient had to be assessed for rigid contact lenses to correct irregular astigmatism.

Six of 100 patients developed symptomatic anisometropia with aniseikonia postoperatively (6%). Four of these subjects decided to proceed with clear lens exchange surgery to correct induced anisometropia despite absence of visually significant cataract (4%). Two initially trialled contact lenses (2%). One was intolerant to their use, while the other later decided for convenience they would prefer to proceed with clear lens exchange. One patient, who was pseudophakic in the operated eye at presentation, but phakic in the contralateral eye, was a CL wearer, and thus, their anisometropia was easily corrected with an up-to-date refraction. Only one patient (1%), who was pseudophakic in both eyes at presentation, had persistent anisometropia/aniseikonia. They decided not to opt for further surgery or to wear contact lenses and utilised their induced myopia for reading (monovision). One patient (myopic) developed a large anisometropia, but because of poor VA and epiretinal membrane with cystoid macular oedema, was not symptomatic. No induced refractive error mandated the removal of the encirclement.

At discharge, nine patients had a BCVA in the operated eye that was severely reduced, being counting fingers or worse. With these patients excluded, mean LogMAR BCVA at discharge or last attendance was 0.49 (SD 0.35; 95% CI 0.41–0.56).

## 4. Discussion

Elongation of AL following encirclement has previously been established and remains clinically important. Induced changes prolong visual rehabilitation, requiring additional refractive correction following surgery. More importantly in a minority, induced anisometropia can prevent simple visual rehabilitation with up-to-date refraction and new spectacles, necessitating further interventions. The number of such occurrences until now remained unreported. As previously demonstrated, AL changes are common following ESB. Published literature varies significantly in terms of surgical techniques, particularly with reference to buckle type and methods of tightening encircling components. With significant shifts in techniques over time, direct comparisons are difficult. Despite numerous papers reporting biometric factors following surgery, to the best of our knowledge none outline changes after encirclement with a broad 276 silicone tire. Furthermore, no reports exist as to the incidence of symptomatic aniseikonia following the encirclement procedure. This seems surprising given the longstanding concerns regarding this issue with reference to corneal and cataract surgery [25]. By contrast, little attention appears to have been paid towards its incidence following vitreoretinal procedures.

In this study, we report the AL changes seen with this technique are comparable to those previously described with narrow silicone encircling bands and localised circumferential scleral buckles, although likely towards the higher end of previously described changes. This is not surprising given previous reports of more extensive buckling being associated with longer AL changes and greater myopic refractive errors [26]. Malukiewicz-Wisniewska demonstrated a mean AL increase at 1 year of 0.57 mm [20], while Vukojevic, using a 1.5 mm encircling band, demonstrated an increase of 0.74 mm [22]. Brazitikos et al. found a mean increase of 0.95 mm [27], while Larsen reports an average elongation of 0.98 mm [15]. Our finding of a mean increase of 1.15 mm is comparable to these previous reports. Past authors have explained the differences in AL changes mainly by the degree of indentation achieved by cerclage tightening, a fact supported by the demonstration that in some instances, with high indentations from encirclement, AL may unexpectedly decrease, particularly if the encirclement causes a string effect [28]. Early eye bank studies with 2 mm encircling bands demonstrated AL increased with low to moderate degrees of indentation, but conversely decreased with high indentations [24], a finding supported by clinical reports [29]. A neglected factor for consideration remains the width of the buckle used for encirclement and the interaction between the two variables. Broad ESB may create more lengthening on average, compared to narrow encircling components, as the sclera may be less likely to invaginate around a wide buckle, resulting in greater lengthening. The influence of scleral invagination anterior and posterior to wide buckles may carry less of an influence compared to the elongation induced from circumferential shortening.

It has been reported that an increase of AL of 1 mm induces a myopic shift of −2 to −2.5 dioptres [24,30]. It would be wrong, however, to extrapolate previous findings and assume any induced anisometropia would be clinically significant in a retinal detachment population, causing symptomatic aniseikonia. Previous reports among cataract populations show the magnitude of surgically induced anisometropia does not always predict degree of aniseikonia [31,32,33].

Previous studies have reported keratometric changes following scleral buckling surgery, indicating corneal curvature changes and induced astigmatism are commonplace [17,19,24,34,35,36,37,38]. Heterogeneity exists in these published reports, reflecting widespread techniques used. Some report that induced astigmatism is slight and transient [24,37,38]. Others report more clinically significant irregular astigmatism [19]. Radial sponge buckles have been associated with significant degrees of astigmatism [19,38]. Hayashi et al. reported all types and combinations of circumferential buckles produce prolonged corneal shape changes with no significant differences in degree of induced astigmatism. The patterns differed, however, depending on procedure, with ESB causing irregular astigmatism if the band is tightened unequally or sutured obliquely [18]. Another comparative study reports that induced astigmatism appears greatest when encirclement is combined with a segmental buckle [39]. In this study, we observed induced astigmatism after encirclement with a broad buckle, but for the majority it was of little clinical significance, with large changes being infrequent. Its average magnitude was less than some previous reports [34] and similar to others [39].

In clinical practice, ophthalmologists manage 6 to 7.6% of anisometropic patients who are at risk of associated aniseikonia following cataract or refractive surgery. A previous large study reported anisometropia as a common finding, with a prevalence as high as 6% in a young population [40]. Among patients with unilateral pseudophakia, Kramer et al. reported an aniseikonia prevalence of up to 10% [31]. High rates of near-vision aniseikonia (up to 12%) were reported for first generation multifocal intraocular lenses [41]. In comparison, far fewer data exist on the incidence of clinically significant aniseikonia following RD surgery. Reports state that, along with reduced vision and metamorphopsia, aniseikonia appears commonly reported, being present in 35% following RD surgery in one questionnaire [42]. Where objective changes in aniseikonia were recorded following successful macular-off retinal detachment surgery, almost half of patients had aniseikonia at 12 months, with the mean being measured at −3.1%, ranging from −9% to +2%. Symptoms were induced when aniseikonia was 3–5% [43]. Approximately 31% of patients had micropsia of 5% or greater at 12 months. Only three patients in this series, however, had buckles, and all were circumferential with no encirclement [43].

Aniseikonia among RD populations is multifactorial, occurring secondary to optically induced anisometropia from interocular differences in refractive status, secondary to factors including silicone oil fill, AL changes from encirclement and myopic shift from cataracts. It can also be induced by retinal changes, and studies suggest that the area of retinal detachment [38,44], the presence of cystoid macular oedema (CMO) and ERM are important aetiological factors [43]. Of RD patients with aniseikonia, 69% exhibit macular structural abnormalities, including ERM, hyper-reflectivities within the ellipsoid zone (EZ), disruption to the EZ, subretinal fluid, macular hole or CMO following RD surgery [44]. Similar degrees of aniseikonia have been reported following pneumatic retinopexy (60%), with measurements ranging from 1% to 4%, with aniseikonia being associated with macular detachment [45]. Importantly, however, the authors reported that most of these patients remained asymptomatic, with aniseikonia only being detected with the New Aniseikonia Test [45].

We report that, within this retinal detachment population, only a minority (6%) developed symptomatic anisometropia with aniseikonia following combined PPV, encirclement and endotamponade, which appeared to be the result of the refractive changes induced by the encirclement. There appears to be a 4% risk of requiring a clear lens exchange to manage induced anisometropia/aniseikonia. The population at risk of requiring more complex surgical interventions other than cataract surgery/clear lens exchange to resolve intractable anisometropia/aniseikonia appears small (1% in this population). Patients with bilateral pseudophakia presenting with a retinal detachment are an at-risk group following encirclement. For the majority, the addition of an encirclement and associated changes in refraction is infrequently associated with symptomatic anisometropia. Visual rehabilitation remains an option through cataract surgery in most for whom it is an issue. Other factors including area of retinal detachment and retinal displacement may be more important aetiological factors [46,47].

Strengths of our study include its large consecutive nature of 100 patients, giving a good representation of possible extreme changes. We use a standardised approach and report results using a single encirclement technique, rather than mixed buckling methods. We describe the encirclement procedure, with a precise objective technique being described, using unambiguous terms and anatomical landmarks, which should encourage repeatable results. We measured AL using the IOLMaster postoperatively and used the contralateral AL as the control. These measurements are more likely to be accurate when compared to those obtained from earlier studies using ultrasonography [22] and those taken preoperatively in the presence of a macular-off RRD, where AL could be significantly underestimated. It is known that within retinal detachment populations, biometry is more accurate using data from the contralateral non-detached eye [48].

The main limitation of this study is that patients are self-reporting symptoms suggestive of anisometropia/aniseikonia. The diagnosis was clinical, with the degree of aniseikonia remaining unquantified using commercially available tests. This is, however, also a strength, as measuring aniseikonia in asymptomatic patients is largely academic. Our figure reflects the number of individuals within an RD cohort who will suffer significantly from changes induced from their PPV and encirclement, which is important for appropriate consenting processes. Measurement and quantification of image sizes between the two eyes, however (using aniseikonia tests), would have added further information regarding asymptomatic subclinical findings.

The retrospective nature of the study is a major limitation and may introduce bias in that not all reports of aniseikonia may have been fully documented or investigated appropriately at the time of clinical attendance. As a tertiary referral centre, we provide emergency vitreoretinal surgery cover for a wide geographic area and refractive outcomes and biometric data following ESB was not universally recorded for all, with discharge back to the original referring units possibly introducing bias. However, follow-up duration was generally significant enough for major symptoms to have been manifest and hopefully recorded, with very few patients being lost to follow up.

There are multiple potential reasons why some of our patients may have had symptoms suggestive of aniseikonia. We acknowledge postoperative aniseikonia is not always secondary to scleral buckling and anisometropia, occurring in patients who have a vitrectomy with gas tamponade or even pneumatic retinopexy [45]. Some may have developed symptoms from changes in retinal microstructure, such as CMO rather than AL changes, or a combination of the two factors. Additional analysis of changes in retinal microstructure and the use of fundus autofluorescence (to identify postoperative displacement of the retina) would be beneficial in attributing causation for aniseikonia with greater certainty.

Unrecognised restriction in eye movements and/or reduced VA may have contributed or worsened symptoms, and it is sometimes impossible to attribute causation. In addition, having repeat biometries in a longitudinal fashion would allow assessment of changes over time which would add invaluable information for subsequent cataract surgery timing.

Corneal topography would be useful, allowing analysis of more peripheral corneal changes, particularly with regards to eyes demonstrating large shifts in keratometry values, given the IOLMaster makes measurements using a central 2.3 mm radius [49]. Explaining why some individuals develop large degrees of astigmatism could have been investigated further by observing the position and degree of the indent achieved among outliers. This would be of interest to assess whether eyes with significant astigmatism have unequal indents in each quadrant, possibly reflecting unequal tightness of mattress sutures or possibly an erroneously placed anterior suture and buckle.

Satisfaction with refractive outcome was not studied, and is beyond the scope of this publication. There may be patients who were pseudophakic and emmetropic who become disappointed at the need for refractive error correction post encirclement. This cohort remains unrecorded within this population.

## 5. Conclusions

In conclusion, AL and corneal curvature changes are induced by encirclement with broad solid silicone buckles similar in extent to those reported using narrow encircling components. For the majority of patients within retinal detachment populations, the degrees of myopia induced appear unlikely to be visually significant or to cause symptomatic anisometropia/aniseikonia. Simple and early visual rehabilitation can often be achieved with refraction and new glasses. Many patients are phakic and develop visually significant cataracts, allowing correction of refractive changes induced with the aim of visual restoration, removing any anisometropia simultaneously. A minority of patients will require more complex and prolonged methods such as contact lens wear or clear lens exchanges. Caution and appropriate consent should be made in emmetropic pseudophakic patients or those with high demands for emmetropic refractive status, such as those with toric intraocular lenses at presentation.

## Figures and Tables

**Table 1:** Summary table for mean biometric data comparing control eyes to postoperative values following combined pars plana vitrectomy (PPV) and encircling scleral buckle surgery. Data are mean ± SD (95% CI).

Variable	Control Eye Value	Postoperative Value	Change	*p*-Value
**Axial length (mm)**	25.39 ± SD 1.27 (24.94–25.83)	26.54 ± 1.16 (26.16–26.91)	1.15 ± 0.67 (0.91–1.39)	0.0001
**Corneal astigmatism (D)**	−0.95 ± 0.51 (−0.78 to −1.13)	−1.33 ± 0.87 (−1.04 to −1.61)	−0.41 D ± 0.86 (−0.11 to −0.71)	0.03
